# Time-Transient Effects of Silver and Copper in the Porous Titanium Dioxide Layer on Antibacterial Properties

**DOI:** 10.3390/jfb11020044

**Published:** 2020-06-22

**Authors:** Masaya Shimabukuro, Akari Hiji, Tomoyo Manaka, Kosuke Nozaki, Peng Chen, Maki Ashida, Yusuke Tsutsumi, Akiko Nagai, Takao Hanawa

**Affiliations:** 1 Department of Biomaterials, Faculty of Dental Science, Kyushu University, 3-1-1 Maidashi, Higashi-ku, Fukuoka 812-8582, Japan; 2 Graduate School of Medical and Dental Sciences, Tokyo Medical and Dental University, 1-5-45 Yushima, Bunkyo-ku, Tokyo 113-8549, Japan; ma190081@tmd.ac.jp (A.H.); manaka.met@tmd.ac.jp (T.M.); k.nozaki.fpro@tmd.ac.jp (K.N.); 3 Institute of Biomaterials and Bioengineering, Tokyo Medical and Dental University, 2-3-10 Kanda-Surugadai, Chiyoda-ku, Tokyo 101-0062, Japan; chen.met@tmd.ac.jp (P.C.); ashida.met@tmd.ac.jp (M.A.); hanawa.met@tmd.ac.jp (T.H.); 4 Research Center for Structural Materials, National Institute for Materials Science (NIMS), 1-2-1 Sengen, Tsukuba, Ibaraki 305-0047, Japan; TSUTSUMI.Yusuke@nims.go.jp; 5 Department of Anatomy, School of Dentistry, Aichi Gakuin University, 1-100 Kusumoto, Chikusa-ku, Nagoya 464-8650, Japan; aknagai@dpc.agu.ac.jp

**Keywords:** antibacterial, surface modification, coatings, implant, biofilm, silver, copper

## Abstract

Recently, silver (Ag) and copper (Cu) have been incorporated into a titanium (Ti) surface to realize their antibacterial property. This study investigated both the durability of the antibacterial effect and the surface change of the Ag- and Cu-incorporated porous titanium dioxide (TiO_2_) layer. Ag- and Cu-incorporated TiO_2_ layers were formed by micro-arc oxidation (MAO) treatment using the electrolyte with Ag and Cu ions. Ag- and Cu-incorporated specimens were incubated in saline during a period of 0–28 days. The changes in both the concentrations and chemical states of the Ag and Cu were characterized using X-ray photoelectron spectroscopy (XPS). The durability of the antibacterial effects against *Escherichia coli* (*E. coli)* were evaluated by the international organization for standardization (ISO) method. As a result, the Ag- and Cu-incorporated porous TiO_2_ layers were formed on a Ti surface by MAO. The chemical state of Ag changed from Ag_2_O to metallic Ag, whilst that of Cu did not change by incubation in saline for up to 28 days. Cu existed as a stable Cu_2_O compound in the TiO_2_ layer during the 28 days of incubation in saline. The concentrations of Ag and Cu were dramatically decreased by incubation for up to 7 days, and remained a slight amount until 28 days. The antibacterial effect of Ag-incorporated specimens diminished, and that of Cu was maintained even after incubation in saline. Our study suggests the importance of the time-transient effects of Ag and Cu on develop their antibacterial effects.

## 1. Introduction

Prosthetic joint infection (PJI) is a devastating and threatening complication for patients and orthopedists [1,2]. Biofilm, which is a main cause of PJI, is the final state of infections. Invaded bacteria generally initiate the infection by adhering onto the implant surface, and grow through a specific mechanism such as extracellular polysaccharide (EPS) production [3,4,5]. The removal of matured biofilms from the implant is difficult because biofilms are resistant to antibiotics due to their bacterial diversity and the presence of the EPS [6,7,8,9]. Often, the only way to eradicate the infection and prevent sepsis is to remove the contaminated device from the patient. To avoid this, biofilm formation must be prevented by inhibiting the initial stage of biofilm formation, namely bacterial invasion, adhesion and growth.

Antibacterial property, which can kill the bacteria, is a necessary and required bio-function on implant surfaces. Silver (Ag) and copper (Cu) are major antibacterial elements, and their effects on various bacteria have been examined by many studies. Ag and Cu potently inhibit various pathogenic bacteria [10,11,12,13,14]. Various surface modification techniques have been used to incorporate these elements on implant surfaces, and their efficacies have been shown by in vitro [15,16] and in vivo [17,18] experiments.

Micro-arc oxidation (MAO), which is an anodic oxidation process with micro-discharges on the specimen surface under high voltage, form a connective porous oxide layer with the incorporation of elements from the electrolyte solution. MAO using the calcium (Ca)- and phosphorous (P)-containing electrolyte improves the hard-tissue compatibility of titanium (Ti), owing to calcium phosphate formation, the promotion of osteoblast adhesion and proliferation, as well as the acceleration of calcification [19,20,21,22,23,24,25,26]. The widespread use of Ti in metallic biomaterials reflects the good mechanical property and biocompatibility of these materials. In addition, some studies have focused on the incorporation of antibacterial elements on Ti surfaces by MAO. Ag- and Cu-incorporated TiO_2_ coatings reportedly exhibit antibacterial activity [27,28,29,30,31,32,33,34,35].

PJI comprises an early infection (within three weeks after surgery) and a late-onset infection (approximately three to eight weeks after surgery), because the bacterial invasion can occur due to implant surgery or the hematogenous spread of bacteria [36,37,38]. Thus, the long-term inhibition of biofilm formation relies on the durability of antibacterial effects. The surface changes are key in the development of antibacterial effect. Therefore, biodegradation behavior must be precisely characterized to understand the antibacterial effect and its durability. However, little attention has been given to the biodegradation behavior of antibacterial coatings. Therefore, the relationship between the surface change and the durability of antibacterial property on Ag and Cu in an in vivo environment is still unknown.

We investigated the long-term behaviors of Ag and Cu in the porous TiO_2_ layer formed by MAO treatment. The changes in both the concentrations and chemical states of the Ag and Cu in the oxide layer incubated for a prolonged period in physiological saline were characterized using X-ray photoelectron spectroscopy (XPS). Moreover, the change of antibacterial property was evaluated using the international organization for standardization (ISO) method with *Escherichia coli* (*E. coli*). In other words, the aim was to clarify the time-transient effects of Ag and Cu in porous TiO_2_ layers on antibacterial property.

## 2. Materials and Methods

### 2.1. Specimen Preparation

Ag- and Cu-incorporated porous titanium dioxide layers were prepared on a commercially pure Ti (grade 2) surface. The ti disks were prepared from the rod of Ti, and each surface was mechanically polished using #320, #320, #600 and #800 silicon carbide abrasive papers. After polishing, all the specimens were washed by ultra-sonication in acetone and ethanol for 10 min.

The electrolyte compositions for MAO were 150 mM calcium acetate and 100 mM calcium glycerophosphate solution, containing 2.5 mM silver nitrate or 2.5 mM copper chloride. The electrochemical parameters were a voltage of 400 V and a current density of 251 Am^−2^, and the treatment time was 10 min. The area in contact with the electrolyte was 39 mm^2^ using the working electrode [39]. After the MAO treatment, all specimens were incubated in a saline during 0 to 28 days. The specimens were fixed onto a polyethylene container to allow the release of metal ions from the surface into the saline. Incubation was performed at 37 °C in a humidified chamber under constant shaking (100 rpm). Every seventh day, the pooled solution was changed with a fresh one. This process simulated a simple biodegradation of Ag- and Cu-incorporated specimens in the body. The specimens after incubation in saline for each period were used further for the surface characterization and evaluation of antibacterial activity. After the MAO and incubation, the surfaces were thoroughly washed in ultrapure water in order to remove any solution remaining in the porous oxide layer.

### 2.2. Surface Characterization

Surface morphologies on the specimens incubated during 0 and 28 days were observed by scanning electron microscopy with energy dispersive X-ray spectrometry (SEM/EDS; S-3400NX, Hitachi High-Technologies Corp., Tokyo, Japan). X-ray diffraction (D8 ADVANCE, Bruker, Billerica, MA, USA) was performed to characterize the crystal structure of each specimen. X-ray photoelectron spectroscopy (XPS; JPS-9010MC, JEOL, Tokyo, Japan) was used in this investigation. The detail of the measurement condition was 10 kV of acceleration voltage, 10 mA of current, MgKα (energy: 1253.6 eV) of X-ray source, and was described in our previous study [40]. The calibration of the binding energy was performed based on C 1s photoelectron energy region peak derived from contaminating carbon (285.0 eV). The integrated intensity of each peak was calculated using Shirley’s method [41]. In addition, a modified Auger parameter (α’) of Ag and Cu, calculated from the Ag 3d_5/2_, Ag M_4_VV, Cu 2p_3/2_ and Cu L_3_VV peaks, was used for the investigation of chemical state changes. According to a method described previously [42], the surface composition on each specimen was calculated using a photoionization cross-section of empirical data [43,44] and theoretically calculated data [45].

### 2.3. Evaluation of Antibacterial Activity

The antibacterial activity was evaluated using *E. coli* (NBRC3972). This evaluation was performed according to the ISO 22196: 2007 method. This experiment was approved by the Pathogenic Organisms Safety Management Committee in Tokyo Medical and Dental University (22012-025c). Luria–Bertani (LB) broth (LB-Medium, MP Biomedicals, Irvine, CA, USA) was used as culture medium, and *E. coli* was cultured in this at 37 °C for 24 h. After culturing, the bacterial density in the suspension of this bacterium strain was measured by ultraviolet–visible spectrometer (UV–vis; V-550, JASCO, Tokyo, Japan), and was adjusted by dilution to be 1.0 × 10^6^ CFUs mL^−1^. The specimens, which were used for this evaluation, were sterilized by immersion in 70% ethanol and washed with ultrapure water. The prepared bacterial suspension was dropped on the surface of each specimen and cultured at 37 °C for 24 h using a sterilized cover plastic film. After 24 h culturing, the suspension of *E. coli* was collected from the specimen surface, and transferred onto nutrient agar plates with dilution. The *E. coli* in collected suspension was cultured on the nutrient agar plates at 37 °C during 24 h. The number of viable bacteria was determined by counting the number of colonies formed on the plates. In this evaluation, the specimen without Ag and Cu was used as the negative control and the specimens with Ag or Cu before incubation were used as the positive control because our previous study revealed that these specimens exhibited antibacterial effects for *E. coli* [27,28].

### 2.4. Statistical Analysis

All values are shown as the means ± standard deviation, and commercial statistical software KaleidaGraph (Synergy Software, Reading, PA) was used for statistical analysis. One-way analysis of variance was used following the multiple comparisons with the Student–Newman–Keuls method to assess the data, and *p* < 0.05 was considered to indicate statistical significance.

## 3. Results

### 3.1. Surface Characterization

**Figure 1:**
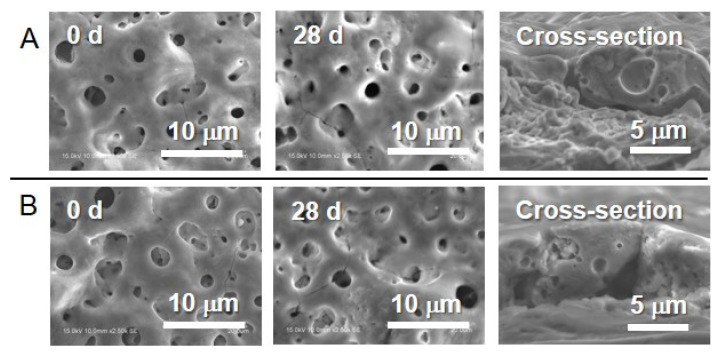
Scanning electron microscopy (SEM) images of (**A**) the Ag- and (**B**) the Cu-incorporated TiO_2_ layers before and after incubation in saline during 28 days and the cross-sectional views of the specimens before incubation.

Typical connective porous morphology after MAO were observed from Ag- and Cu-incorporated TiO_2_ layers. This morphology on each specimen was maintained after incubation in saline during 28 days (Figure 1).

**Figure 2:**
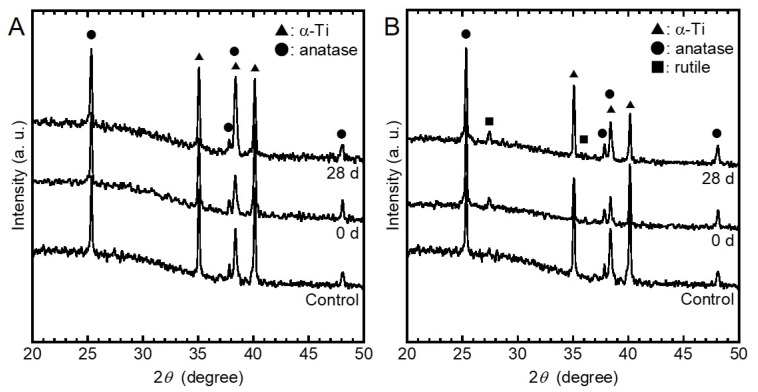
X-ray diffraction (XRD) spectra obtained from the (**A**) Ag- and (**B**) the Cu-incorporated specimens before and after incubation in saline during 28 days. The spectra presented at the bottom in each figure was obtained from the control.

The XRD spectra obtained from the control, the specimens before incubation, and the specimens after incubation are presented in Figure 2, respectively, from the bottom to the top in each figure. Peaks corresponding to α-Ti and anatase TiO_2_ were detected, and those of Ag were undetected in the Ag-incorporated specimens before and after incubation. Peaks corresponding to α-Ti, anatase TiO_2_, and rutile TiO_2_ were detected, while those of Cu were undetected. Furthermore, the chemical structures of Ag- and Cu-incorporated specimens did not change by the incubation in saline during 28 days.

**Figure 3:**
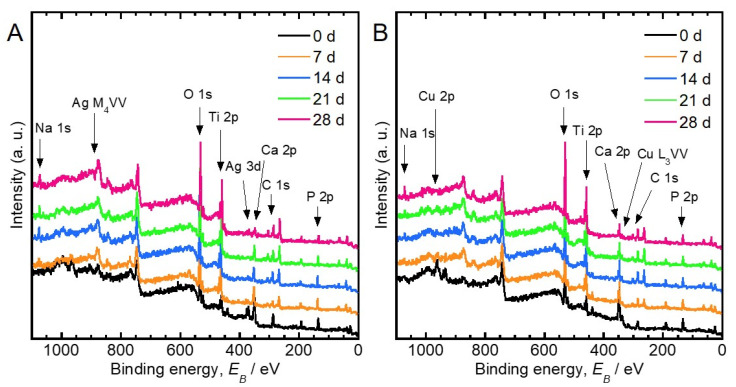
X-ray photoelectron spectroscopy (XPS) survey scan spectra obtained from the (**A**) Ag- and (**B**) the Cu-incorporated specimens before and after incubation in saline during 28 days.

Figure 3 shows the XPS survey scan spectra obtained from Ag- and Cu-incorporated specimens before and after incubation in saline during 28 days. The peaks originating from C, O, P, Ca, Ti, and Ag or Cu were detected from the XPS spectra of the Ag- and Cu-incorporated specimens. In addition to these elements, the peak originating from Na was detected in the specimens after incubation in saline from 7 to 28 days. P existed as a phosphate species and calcium existed as Ca^2+^, because the binding energies of the corresponding peaks of P 2p and Ca 2p_3/2_ were 133.7–134.1 eV and 347.6–347.9 eV, respectively. The binding energy of the Ti 2p_3/2_ peak was 458.9–459.3 eV, indicating that Ti existed as TiO_2_. The binding energies of the Na 1s peaks were 1071.7–1072.4 eV, indicating that Na exists as Na^+^.

**Figure 4:**
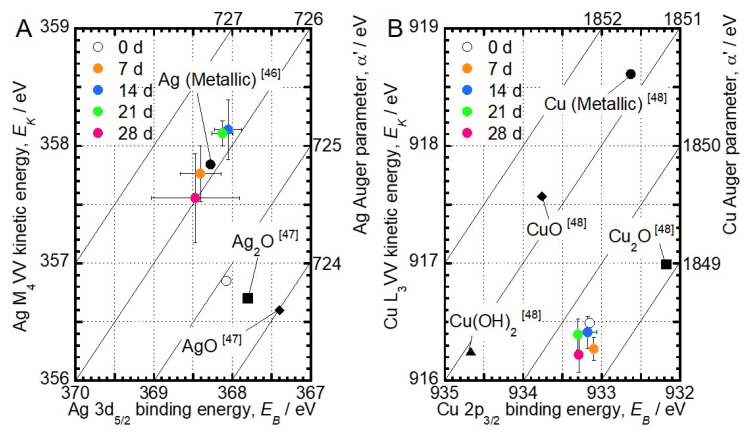
Wagner plot of (**A**) the Ag and (**B**) the Cu in the oxide layer incubated in saline for 28 days based on the photoelectron peaks and the Auger peaks. Each parameter of the Ag and the Cu compounds is plotted according to the previous studies [46,47,48].

Figure 4 depicts the Wagner plot of Ag and Cu obtained from this XPS characterization and previous studies [46,47,48]. The chemical state change of Ag and Cu is determined by the comparison of their binding energy with α’ values on the Wagner plot. According to the Wagner plot of Ag, the α’ value of the Ag-incorporated specimen before incubation (0 days) was 724.9 eV, indicating that Ag mainly exists as Ag_2_O. The α’ values of Ag increased with incubation time. Since the α’ value finally converged to 726.1 eV, it became clear that the chemical state of Ag incorporated in TiO_2_ by MAO approached that of metallic Ag with increasing incubation times. On the other hand, the α’ values of Cu from the specimens immersed in saline for 28 days were 1849.3–1849.7 eV, indicating that Cu exists as Cu_2_O. The chemical state of Cu in the oxide layer did not change during the incubation in saline.

**Figure 5:**
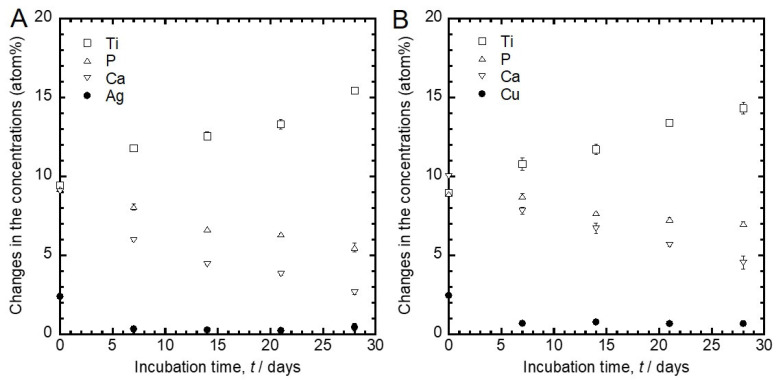
Changes in the atomic concentrations in (**A**) the Ag- and (**B**) the Cu-incorporated TiO_2_ layers with the incubation time.

Figure 5 shows the changes in the concentrations of Ti, P, Ca and Ag or Cu detected from Figure 5A’s Ag- and Figure 5B’s Cu-incorporated specimens with the incubation time. The concentrations of Ag and Cu were relatively small (around 2.5 atom%), even before the incubation. Furthermore, from the results of the EDS analysis of the cross-section shown in Figure 1, 0.1 atom% of Ag and 0.1 atom% of Cu were detected from the inside oxide layers, respectively. Thus, the amounts of Ag and Cu incorporated during the MAO treatment were small. The amount of Ag and Cu dramatically decreased to approximately Ag 0.4 atom% and Cu 0.8 atom% upon incubation in saline from 0 to 7 days, respectively. These concentrations remained constant until 28 days. Moreover, the concentrations of Ca and P decreased, and that of Ti increased with the incubation time.

### 3.2. Evaluation of Antibacterial Activity

**Figure 6:**
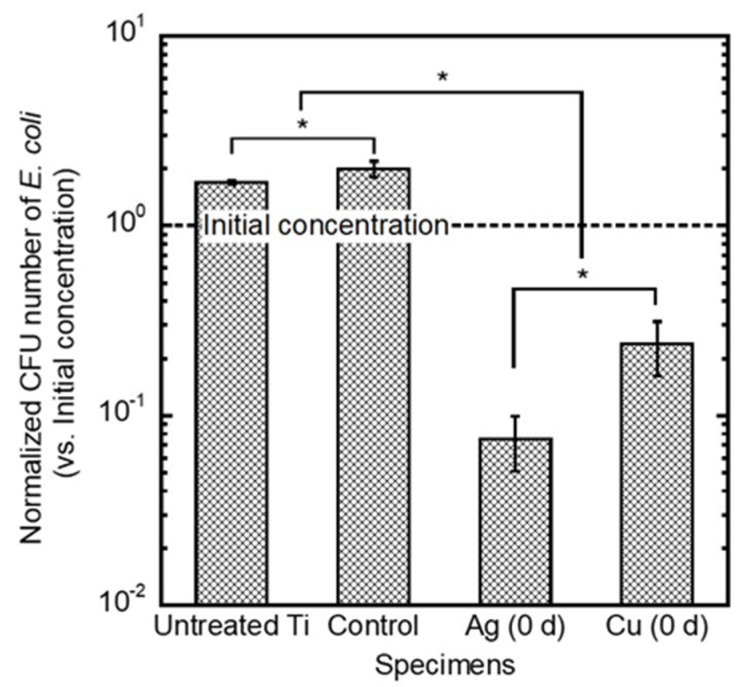
Comparison of the antibacterial effects of the untreated Ti control specimen (micro-arc oxidation (MAO)-treated Ti without antibacterial elements), the Ag- and the Cu-incorporated specimens. Data are shown as the mean ± standard deviation. * Significant difference between specimens (*p* < 0.05).

The normalized bacterial number of *E. coli* on each specimen is shown in Figure 6. The vertical axis represents the bacterial number normalized by the initial concentration of *E. coli*. The normalized bacterial number smaller than 1 (shown as a dashed line in the figure) indicates that the tested specimens exhibited an antibacterial effect. *E. coli* grew on the untreated Ti and control specimen, because the number of *E. coli* on those specimens significantly increased compared with the initial bacterial number. In contrast, Ag- and Cu-incorporated specimens developed antibacterial effects against *E. coli*.

**Figure 7:**
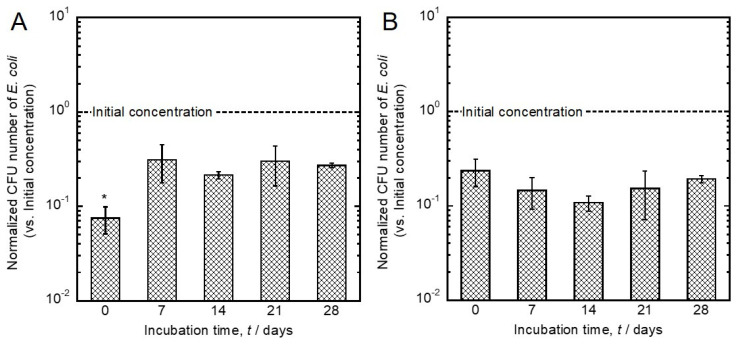
Changes of the antibacterial effects of (**A**) the Ag- and (**B**) the Cu-incorporated specimens before and after incubation in saline. Data are shown as the mean ± SD. * Significant difference between the specimens before and after incubation (*p* < 0.05).

Changes in the antibacterial effects of Ag- and Cu-incorporated specimens before and after incubation in saline for 28 days are shown in Figure 7. The antibacterial effect of the Ag-incorporated specimen after incubation was significantly weakened compared to that before incubation. This effect was at the same level as that of Cu-incorporated specimens. On the other hand, the antibacterial effects of Cu-incorporated specimens did not change upon incubation, and were maintained even after the 28-day incubation in saline.

## 4. Discussion

The porous oxide layers formed by MAO did not change their surface morphology and crystal structure during the incubation in saline (Figure 1 and Figure 2). The lack of change is highly beneficial for the development of antibacterial property on implant surfaces.

The chemical states of P, Ca, and Ti in the Ag- and Cu-incorporated oxide layers were phosphate, Ca^2+^, and TiO_2_, respectively. These chemical states did not change upon incubation in saline for up to 28 days (Figure 3). The porous oxide layer consisted of TiO_2_ as well as incorporated Ca, P, and antibacterial elements (Figure 3 and Figure 5). The presence of Ca and P in the porous oxide layer makes the hard-tissue compatibility of Ti better [27]. The peak originating from Na^+^ was detected from the specimens after incubation in saline for 7 to 28 days. It is conceivable that compounds related to Na^+^ were generated on the specimen surfaces, owing to the interfacial reactions between oxide layer and saline.

Ag- and Cu-incorporated porous oxide layers were formed on Ti surfaces, and slight amounts of Ag and Cu were incorporated by MAO using the electrolyte containing Ag and Cu (Figure 1 and Figure 5). These results indicate that the constituent elements in the electrolyte were incorporated into the porous oxide layer during the MAO treatment. The incorporations of Ag and Cu by MAO is beneficial for realizing the antibacterial property on the implant surface (Figure 6). In addition, our previous studies revealed that specimens with Cu and a suitable amount of Ag did not affect the cellular adhesion, proliferation, differentiation or the calcification of the osteoblast cells [27,28]. Therefore, MAO can be imparting dual-function to the Ti surface, namely antibacterial property and hard-tissue compatibility.

The concentration of Ag in the oxide layer was dramatically decreased, and the chemical state of Ag in the oxide layer was changed from Ag_2_O to metallic Ag during the incubation in saline (Figure 4 and Figure 5). These results indicate that Ag_2_O was converted into chemically stable metallic Ag in saline, due to the release of Ag ions. A study that investigated the formation mechanism of Ag particles in sodium citrate solution described the reduction of Ag on Ag particles via the radical-to-particle electron transfer [49]. The α’ values of Ag in the oxide layer increased with incubation time, indicating that the density of electrons increased. Therefore, like the reduction of Ag on Ag particles in the solution containing sodium citrate, the change of the chemical state of Ag in the oxide layer incubated in saline may be caused by radical-to-Ag electron transfer. Moreover, the antibacterial effects of Ag-incorporated specimens changed upon incubation in saline (Figure 7). This finding implicates changes of both the concentration and the chemical state of Ag in the oxide layer in the antibacterial effect. However, it can be considered that Ag changes its chemical state more drastically depending on components, such as sulfur, in the actual biological environment, since Ag changed its chemical state, even in the simple simulated body fluid. Therefore, changes in the chemical state of Ag in the actual biological environment could influence the antibacterial effect.

The concentration of Cu in the oxide layer was dramatically decreased, and the chemical state of Cu in the oxide layer did not change upon incubation in saline (Figure 4 and Figure 5). The chemical state of Cu in the oxide layer was stabilized as Cu_2_O despite the difference in incubation time. This result indicates that the Cu_2_O is a stable chemical state in the TiO_2_ layer. In addition, the antibacterial effect of the Cu-incorporated specimen was maintained even after the 28 days of incubation in saline. These results indicate that Cu_2_O in a stable chemical state has a more important role in the development of an antibacterial effect, compared with the change of surface concentration of Cu. Previous studies revealed a substantial difference in antibacterial effects between CuO and Cu_2_O. CuO inhibited the development of an antibacterial effect compared to metallic Cu. In contrast, thermally generated Cu_2_O was as effective as metallic Cu [50,51]. In other words, the presence of Cu_2_O and the slight amount of Cu in the TiO_2_ layer are necessary to develop the antibacterial effect.

The results support the proposal that the concentrations of Ag and Cu in the oxide layer are easily and dramatically changed by the incubation in saline. In addition, the chemical state of Ag changed from Ag_2_O to metallic Ag, while that of Cu did not change. The antibacterial effect of an Ag-incorporated specimen for Gram-negative bacteria changed by the 28-day incubation in saline, while the activity of Cu was maintained. The collective findings indicate the importance of the time-transient effects of Ag and Cu. This knowledge will be useful in the design of antibacterial implants based on the surface changes of Ag and Cu in vivo. Further study will be necessary to reveal the long-term effects of Ag and Cu for Gram-positive bacteria.

## 5. Conclusions

Ag- and Cu-incorporated porous titanium dioxide layers were formed on Ti surfaces by MAO treatment. The chemical states of P, Ca, and Ti in the Ag- and Cu-incorporated oxide layer were phosphate, Ca^2+^, and TiO2, respectively. These chemical states did not change upon incubation in saline for 28 days. Moreover, the chemical state of Ag changed from Ag_2_O to metallic Ag, and that of Cu did not change by incubation in saline for up to 28 days. The concentrations of Ag and Cu were dramatically decreased by incubation for up to 7 days, and remained a slight amount until 28 days. The antibacterial effect of Ag-incorporated specimens changed, and the effect of Cu was maintained even after incubation in saline. The findings highlight the importance of the time-transient effects of antibacterial elements on their antibacterial properties.
